# Characterization of histone modification patterns and prediction of novel promoters using functional principal component analysis

**DOI:** 10.1371/journal.pone.0233630

**Published:** 2020-05-27

**Authors:** Mijeong Kim, Shili Lin

**Affiliations:** 1 Department of Statistics, Ewha Womans University, Seoul, Republic of Korea; 2 Department of Statistics, Ohio State University, Columbus, Ohio, United States of America; George Mason University, UNITED STATES

## Abstract

Characterization of distinct histone methylation and acetylation binding patterns in promoters and prediction of novel regulatory regions remains an important area of genomic research, as it is hypothesized that distinct chromatin signatures may specify unique genomic functions. However, methods that have been proposed in the literature are either descriptive in nature or are fully parametric and hence more restrictive in pattern discovery. In this article, we propose a two-step non-parametric statistical inference procedure to characterize unique histone modification patterns and apply it to analyzing the binding patterns of four histone marks, H3K4me2, H3K4me3, H3K9ac, and H4K20me1, in human B-lymphoblastoid cells. In the first step, we used a functional principal component analysis method to represent the concatenated binding patterns of these four histone marks around the transcription start sites as smooth curves. In the second step, we clustered these curves to reveal several unique classes of binding patterns. These uncovered patterns were used in turn to scan the whole-genome to predict novel and alternative promoters. Our analyses show that there are three distinct promoter binding patterns of active genes. Further, 19654 regions not within known gene promoters were found to overlap with human ESTs, CpG islands, or common SNPs, indicative of their potential role in gene regulation, including being potential novel promoter regions.

## Introduction

The importance of characterizing histone modification patterns in the promoter regions has been elucidated in Heintzman et al. [1] and other research after this seminal publication [2–7]. Histones are proteins, found in the cell nucleus, on which DNA is wrapped around; they are epigenetic marks that play an important role in gene expression regulation. A common form of histone modification is histone methylation, in which one, two, or three methyl groups may be transferred to lysine. For example, the mono-, di-, ad tri-methylation of lysine 4 on histone H3, denoted as H3K4me1, H3K4me2, and H3K4me3, are the addition of one, two, or three methyl groups, respectively. Histone acetylation is another common form of histone modification (in which lysine residues are acetylated) and is also essential for gene regulation.

Modifications (predominantly enrichment) of a number of histone marks have been observed in the promoter regions around the transcription start sites (TSS) [8–10]. In particular, H3K4me2 and H3K4me3 have been found to be associated with transcriptional activation, with increased level in the promoter region of activate genes [2, 7, 11–15]. Similarly, there is also evidence showing that active genes are characterized by high level of acetylation of the H3K9, H3K9ac [15–19]. On the other hand, the monomethylated H4K20me1 has been found to be associated with gene silencing, although there are recent studies that indicate that H4K20me1 is enriched in active gene promoters or gene body [10, 20–23], and increased level of H4K20me1 on the X chromosome has also been observed [24].

The large body of research on the characterization of histone marks on the promoter regions indicate that histone modification patterns are dynamic, can be perturbed in diseased cells, and are generally heterogeneous in the population of gene promoters. In fact, distinct chromatin signatures may specify unique genomic functions and thus signify the distinctive features of different cell types [1, 9, 25–27]. Nevertheless, inconsistencies may occur among different studies [14, 16, 19, 28], warranting further analysis to clarify existing and reveal yet to be discovered patterns. Indeed, despite the great progress in whole-genome characterization, novel and alternative promoters, and novel genes continued to be identified in further studies [29–31].

To characterize distinct chromatin signatures and to identify novel and alternative promoters, the combinatorial modification patterns of multiple histones have been studied [1, 5, 29]. Specifically, Heintzman et al. [1] presented a methodology in which promoter region modification patterns of several histones obtained from ChIP-chip experiments were clustered using the non-parametric k-means algorithm. Four distinct classes of promoter modification patterns were observed, in which one cluster has little activities, whereas the other three have different level of increased activities around the TSS with similar patterns of peaks and valleys. These results were taken to imply the confirmation of previous research, which observed the linkage between histone modifications and promoter activity. In an attempt to provide a more refined computational algorithm to better distinguish and characterize the different chromatin modification patterns around the promoters, Taslim et al. [29] proposed a follow-up step after the k-means clustering to fit a fully parametric model to capture the combinations of uni-modal and bi-modal patterns. In a more recent contribution [5] based on a prior notion that histone modification in active regulatory regions follow a peak-valley-peak (PVP) patterns (i.e. bi-modal using the terminology of Taslim et al. [29]), an algorithm was developed to delineate the PVP patterns of transcriptional activities. Patterns identified were then used to identify novel regulatory regions, including novel and alternative promoters [1, 5, 29].

The novelty and scientific importance notwithstanding, some of the existing methods for characterizing combinatorial histone modification patterns do not fully utilize information in the data and are susceptible to a large degree of noise. For example, the k-means approach dealt with potentially noisy large-dimensional data directly and did not account for spatial correlations of nearby modification signals. Other methods used for histone modification pattern characterization are too restrictive, such as assuming a PVP pattern or a fully parametric mixture model. On the other hand, denoising methods such as functional principal component analysis (FPCA) for dimension reduction and signal extraction have been proposed and investigated in the statistical literature and have been adopted to address problems in epigenetics. For example, the individual scores from the Karhunen-Loéve (KL) expansion of FPCA have been used to study correlated variability of pairs of epigenetic datasets [32]. Other modern dimension-reduction methods have also been increasingly used in genomic analysis, such as the application of a non-negative matrix factorization (NMF) approach for classifying the epigenome [33]. However, we note that while FPCA accounts for spatial correlation, NMF typically does not, and furthermore, scores from the KL expansion have nice properties that can be used for further analysis.

To make full usage of available data and to recover the underlying unique patterns without being restricted by any prior notion or masked by noise, in this paper, we propose a novel two-step approach to uncover underlying patterns in a heterogeneous setting. In particular, we focus on the combinatorial patterns of multiple histone modification marks, which has motivated this research. The first step uses a functional principal component analysis method for dimension reduction and signal extraction, which takes spatial correlation into consideration. However, unlike [32], where the scores from the KL expansion were used directly to compare pairs of datasets, in this paper, we make use of the uncorrelated nature of the principal components to carry out the second step of the analysis to study pattern heterogeneity in a population of genes. Specifically, a mixture of normal distributions is formulated to cluster the extracted smooth curves around known transcription starts sites (TSS) where parameters are estimated based on an expectation-maximization (EM) algorithm. As an additional illustration of the utility of the underlying patterns, we use them to scan the entire genome to find novel and alternative promoters.

## Materials and methods

### Data sets

**Fig 1:**
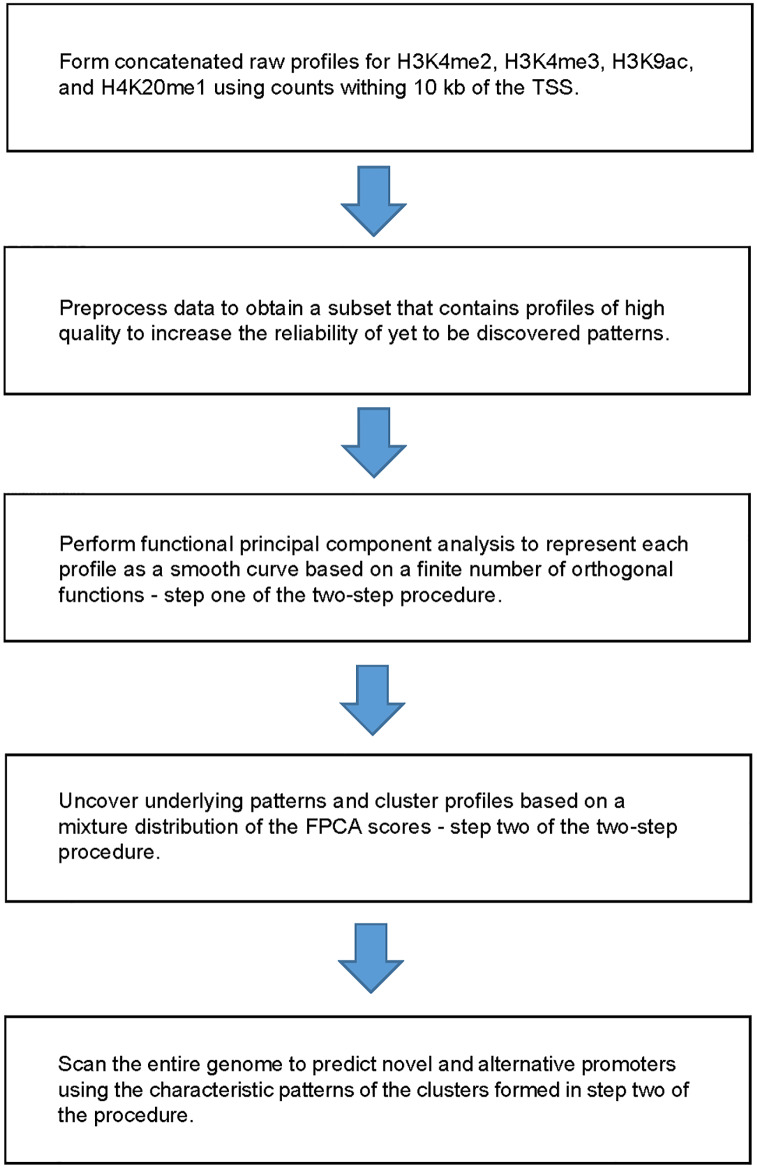
Analysis pipeline for uncovering distinctive characteristic patterns of histone marks and prediction of novel promoters.

We used the human B-lymphoblastoid cell line (GM12878) ChIP-seq whole-genome chromatin modification data [34], which was available from the NCBI Gene Expression Omnibus (GEO) (https://www.ncbi.nlm.nih.gov/geo/query/acc.cgi?acc=GSE26320). The human B-lymphoblastoid cell line was derived from the peripheral blood B lymphocytes, which can be easily cultured and is widely used for studying various aspects of pathology and biology. In particular, we downloaded the histone modification data for H3K4me2, H3K4me3, H3K9ac, and H4K20me1 to characterize their modification patterns around the TSS of known genes. The reason for their selection is that these histone marks have been shown to be associated with increased modification levels in the promoter regions of active gene [9, 35, 36], although, as we have pointed out earlier, there are inconsistency regarding the level of H4K20me1. Studies of various cell lines have shown that H4K20me1 may not necessarily have increased level in the promoter of active genes or only have increased level in the gene body [8, 37, 38]. However, the relationship between H4K20me1 and gene transcription remains controversial. As such, it is of particular interest to study the modification patterns of H4K20me1 in the B-lymphoblastoid cells using an unbiased, robust, yet efficient approach. Our analysis pipeline, which contains a two-step procedure as its centerpiece, is described in the following subsections and summarized in Fig 1.

### Data preprocessing

We followed the procedure in Heintzman et al. [1] and Taslim et al. [29] to process the data. The goal is to obtain a set that contains data of high quality so that there is a greater reliability of the uncovered patterns. Specifically, the modification levels for each of the four histone marks in a 10 kilobase pair (kb) region centered at the TSS of each known RefSeq gene were extracted. We quantified the data into consecutive, non-overlapping bins of 100 base pairs (bp’s); thus, the levels of histone mark in a promoter region was represented by 100 numbers, referred to as intensity counts, 50 upstream and 50 downstream of the TSS. To understand the combinatorial patterns of these four histone modifications, we concatenated these four 10 kb regions into a profile composed of observations, *y*(*t*), on 400 discrete locations {1, 2, …, 400}, for each promoter. In other words, the modification levels in the 100 bins for each of the four histone marks, H3K4me2, H3K4me3, H3K9ac, and H4K20me1, are concatenated in that order, such that *y*(*t*), *t* ∈ {1, 2, …, 100} denote the data from H3K4me2, *y*(*t*), *t* ∈ {101, 102, …, 200} denote the data from H3K4me3, and so forth (S1 Fig provides a schematic diagram). For quality control to consider only promoters for which the modification profiles over all four histone marks are not simply random variation, we excluded those for which the maximum intensity count is less than seven. We further exclude overlapping promoters due to ambiguity of the contribution to the intensity counts following Taslim et al. [29]. This cleaning process has led to a total of 2926 promoter regions for use in characterizing the modification patterns.

For detecting novel and alternative promoters, we formed bins of 100 bp’s and obtained the intensity count for each bin. A scanning approach was taken over each 10 kb window, with the focused window formed by sliding one bin (100 bp) at a time; a modification profile was then formed by concatenating over the four histone marks in the same order as described above. To be consistent, we only considered testing a 10 kb region as a potential novel/alternative promoter if the maximum intensity count over the entire profile was greater than seven. Further, we did not consider any 10 kb region if it overlaps with a known 10 kb promoter region as defined above.

### Characterization of histone modification patterns

For the modification profile of each histone at a known promoter region, the intensity counts over the 100 bins are not independent; rather, they are spatially correlated. Thus, the intensity counts are considered as functional data, because they can be treated as realizations at discrete locations from an underlying smooth curve in the region. Therefore, we adopt a functional data analysis method, FPCA, for dimension reduction; then, we cluster the profiles using an estimated mixture model. More specifically, we propose a two-step procedure that goes beyond the use of the FPCA methodology. In the first step, we perform functional principal component analysis to reduce the dimension of the data by representing the observed intensity counts for each profile as a smooth curve using a finite number of basis functions. In the second step, making use of the uncorrelated property of the FPCA scores (“coefficients” of the basis functions), we formulate a mixture modeling approach to cluster the smooth curves to reveal the underlying combinatorial modification patterns over all four histones. In the following two subsections, we describe each of these two steps in details.

#### Step 1—Functional principal component analysis

FPCA is a frequently employed method for dimension reduction of functional data, as those representing the modification profiles in this application. Although other methods for dimension reduction of functional data are also available, we choose to use FPCA for our specific problem because our data may present narrow bi-modal peaks patterns [5, 29], which may not be captured properly by methods such as B-spline or Fourier transformation [39]. Nevertheless, we note that FPCA is a non-parametric procedure, we do not pre-specify any functional forms. We also note that there are other frequently used dimension reduction methods in genomic research, such as non-negative matrix factorization, but we choose to use FPCA as it accommodates spatial correlation among counts in the region. Finally, since we have observed intensity counts in all bins, this represent a dense data scenario, for which FPCA methods are well developed [40].

Treating our spatially correlated intensity counts data as the discretized observations from a continuous stochastic process {*Y*(*t*), *t* ∈ [0, *T*]} in a continuous time interval, where *T* = 400, we can write it according to Karhunen-Loéve expansion [41, 42] as follows.

$$\begin{array}{c} Y\left(t\right)=\mu \left(t\right)+\sum_{j=1}^{\infty }C_{j}\psi_{j}\left(t\right),\;\;t\in \left[0,T\right], \end{array}$$

where *μ*(*t*) is the overall mean, the *ψ*_*j*_(*t*)’s are orthogonal continuous real-valued functions, and the *C*_*j*_’s are uncorrelated zero-mean random variables and are referred to as principal components (PCs) or scores. For dimension reduction, we only used the first *q* components corresponding to the *q* largest eigenvalues. We used the ‘FPCA’ function of the R package ‘fdapace’ [43] for carrying out this analysis. This leads to the capturing of concatenated “smoothed” profiles for the four histone marks. That is, at the conclusion of the first step, each concatenated profile of the histone marks is represented by a smooth curve expressed as a finite number of combination of the orthogonal basis functions. We will then take this one step further by modeling the scores (the coefficients of the basis functions) to uncover the underlying patterns among the smooth curves, the second-step of our procedure, as described in the following.

#### Step 2—Uncovering underlying patterns and clustering of profiles

With the smooth profiles from FPCA, we are now ready to cluster them to reveal the underlying combinatorial patterns. We assume that there are *K* patterns (i.e. *K* clusters of profiles), where *K* is in fact unknown. Exploiting the property that the PCs are uncorrelated and further assuming that the stochastic process *Y*(*t*) is Gaussian (after log-transformation), then the first *q* PC vector, $C_{q}={\left[C_{1},C_{2},\ldots ,C_{q}\right]}^{T}$, follows a mixture distribution with its density function specified as

$$\begin{array}{c} f\left(C_{q}\right)=\sum_{k=1}^{K}\pi_{k}\prod_{j=1}^{q}f_{j}^{\left(k\right)}\left(C_{j}\right), \end{array}$$

where $f_{j}^{\left(k\right)}\left(C_{j}\right)$ is the univariate Gaussian density function for the *j*^*th*^ PC in the *k*th cluster [39]. Further, *π*_*k*_ is the component weight, which may be interpreted as the a priori probability that a profile belongs to the *k*th cluster. For a fixed *K*, we use the ‘FClust’ function in the ‘fdapace’ R package [43], which calls another R package ‘EMCluster’ that implemented an EM algorithm for clustering of finite mixture Gaussian models.

For our analysis, we fit the model with a range of *K* values, including *K* = 4, which matches the number of promoter region histone modification patterns uncovered in earlier studies [1, 29]. For each of the *K* considered, we examine the profiles that are assigned to each of the *K* clusters. We then choose the optimum *K* to be the smallest such number so that the profiles within each cluster are homogeneous with a clear overall pattern. As discussed in more detail in the results section for the analysis of the dataset, we can see that, when setting *K* = 4, the profiles in each cluster appear to be homogeneous with a clear overall pattern, and the patterns are distinctive and interpretable from cluster to cluster. However, when *K* is set to be larger, one can clearly see that some of the clusters in the *K* = 4 scenario get split into multiple clusters. On the other hand, when *K* is set to be smaller, the patterns are no longer clear. After the appropriate *K* is chosen, the characteristic histone modification pattern (or simply referred to as characteristic pattern hereafter) for each cluster is taken to be the mean of all profiles belonging to that cluster.

### Prediction of novel/alternative promoters

We devise and implement a criterion for predicting putative novel or alternative promoters with high confidence. For each of the profiles in a cluster, we calculated its Pearson’s correlation with the characteristic pattern of the cluster. For the profiles whose contributions to the formation of the characteristic patterns are minimal, their correlations are necessarily small. Therefore, to identify putative promoters genome-wide that follow the characteristic patterns to a suitable extent, we find the 100*α* percentile of the empirical distribution of the correlations for each cluster. A 10-kb test region is deemed to be part of a potential novel/alternative promoter region belonging to a particular cluster, referred to as a *significance window*, if its correlation with the characteristic pattern of that cluster exceeds the 100*α* threshold. It is apparent that a higher *α* level will lead to a higher confidence in a potential novel promoter, but the number of identifications will be smaller. In our analysis, we have chose *α* = 0.4 as a compromise between the sensitivity of uncovering promoters and the level of confidence; we discuss this further in the Discussion section.

**Fig 2:**
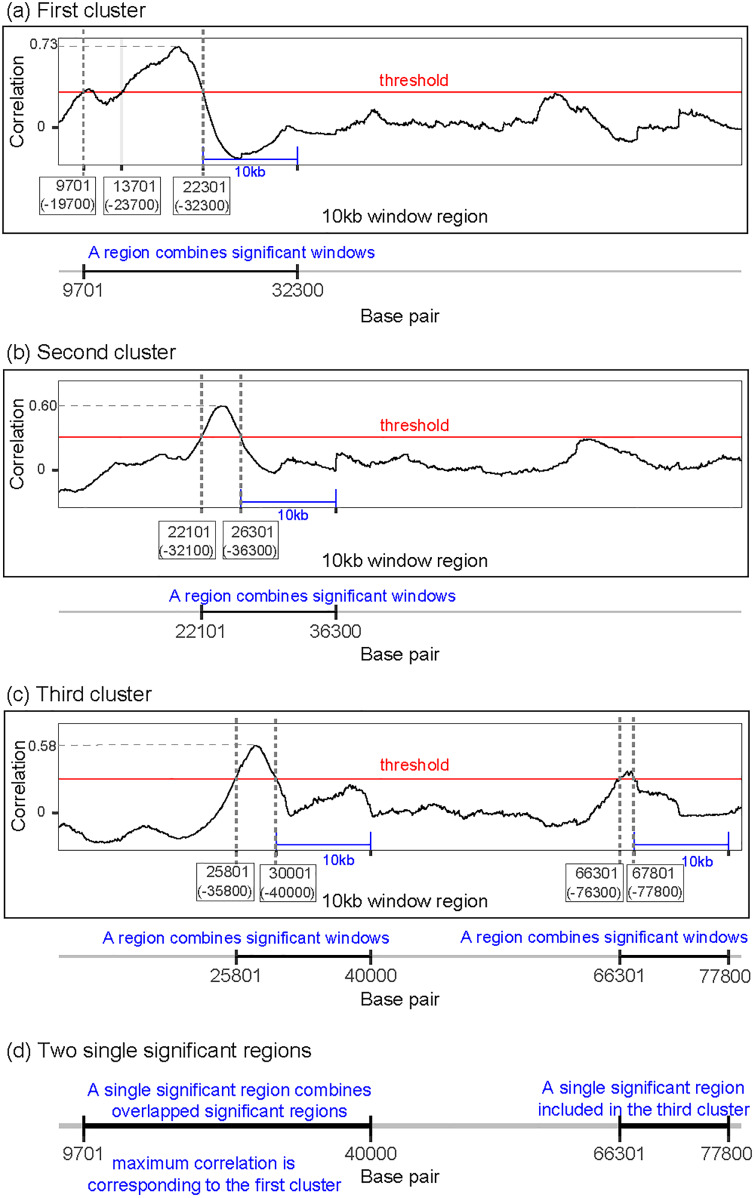
Example of the prediction procedure leading to the identifications of two putative promoter regions. In each of the subplots (A)-(C), the first number in each box indicates and marks the beginning of a 10 kb window; the second number (within the parentheses) indicates the ending of the window, which marks the position 10 kb downstream as drawn. (A) First cluster: a single region (9701 bp—32300 bp) having a significant correlation with cluster 1 characteristic binding pattern. (B) Second cluster: a single region (22101 bp—36300 bp) having a significant correlation with cluster 2 characteristic binding pattern. (C) Third cluster: two regions (25801 bp—40000 bp and 66301 bp—77800 bp) each having a significant correlation with cluster 3 characteristic binding pattern. (D) Two putative promoter regions by combining the results in (A)-(C) according to the rule described.

We adopt a moving-window approach to scan for putative promoters. Each window is composed of 100 bins (each covering 100 bp’s as we discussed earlier) for each histone mark; therefore, the number of bins for computing the correlation is over 400 numbers (4 histones data concatenated in the same order as the characteristic curve). Our focused window moves one bin at a time. Therefore, two consecutive windows share 99 bins for each histone mark, and thus, the computed correlations with a cluster’s characteristic pattern for such windows are highly correlated themselves. Hence, we group overlapping significance windows for a particular cluster into a single *significance region*, as we explain using the example in Fig 2. In Fig 2A, the 10kb window starting at 9701 bp and ending at 19700 bp has a significant correlation with the characteristic curve of the first cluster. The next 10kb window starting at 9801 bp and ending at 19800 bp is also correlated. In this way, we can combine all overlapped significance windows into a significance region that starts at 9701 and ends at 32300 for correlation with the first cluster characteristic binding pattern. Similarly, in Fig 2B, the region starting at 22101 bp and ending at 36300 bp is determined to be significant for correlation with the second cluster binding pattern. In Fig 2C, for the third cluster, the region from 25801 bp to 40000 bp is significantly correlated with the characteristic binding pattern. Furthermore, within the genomic region shown in Fig 2, 66301 bp—77800 bp constitutes another significance region.

Because significance regions arising from different clusters may overlap as we have seen in Fig 2A–2C, we further devise a procedure to identify unique putative promoter regions that only belong to a single cluster. First, we group all overlapping significance regions from all clusters into a single set. For each such set, we choose its cluster membership to be the one that has the largest highest correlations among the clusters. Then the union of the regions in this set is taken to be a putative promoter region belonging to the corresponding cluster. Fig 2D shows that the region from 9701 bp—40000 bp is the union of all significance regions from all three clusters. Since the highest correlation in cluster 1 is larger than those for the other two clusters, this region is therefore classified as a putative promoter region belong to cluster 1. The second putative promoter region shown in the figure, from 66301 bp—77801 bp, belongs to cluster 3, since this is the only cluster (among all three) where the characteristic pattern is significantly correlated with the data in this region.

## Results

### Number of clusters and characteristic patterns

**Fig 3:**
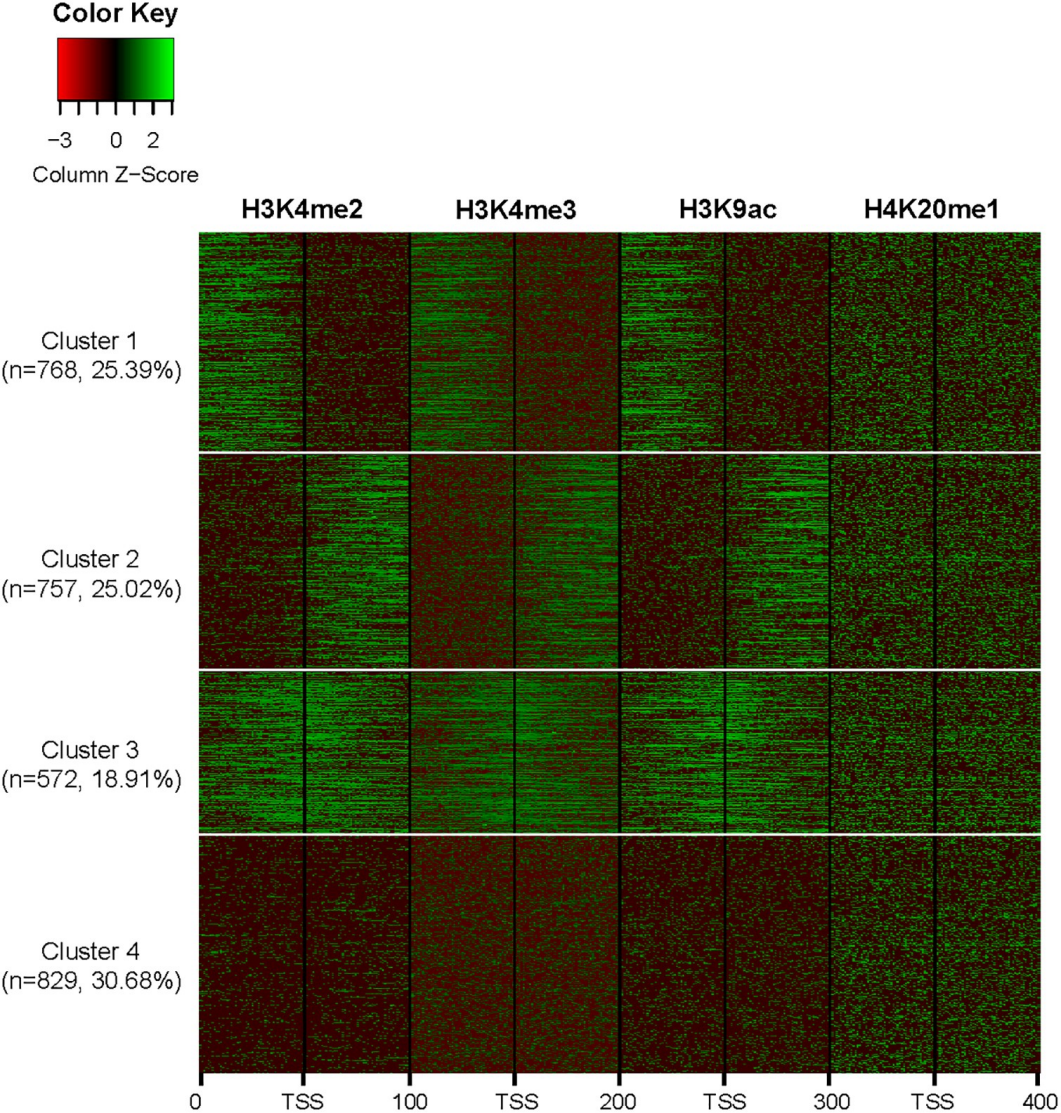
Heatmap of clusters for *K* = 4. Each row represents one profile over all four histone marks, where we can see that cluster 4 is the largest as it has more profiles compared to the other clusters. Plotted are the color representation of the z-scores of the log-transformed intensity counts.

For dimension reduction to better handle noise, we chose seven functional principal components (i.e. *q* = 7) that account for 90% of the variance in the data. After the intensity count data over all four histones are represented as smooth curves using FPCA, we clustered them to uncover the underlying histone modification patterns. We considered the number of clusters *K* = 2 to 6 in increment of 1. For *K* = 4, one can see that the profile curves in each cluster appear to be homogeneous (Fig 3), yet the profiles are distinguishable between clusters. The number of profiles belonging to clusters 1-4 are 768, 757, 572, 829, respectively. For *K* = 5, we see that two of the clusters have similar patterns, indicating an artificial split of one of the *K* = 4 clusters into two (S4 Fig). We see artificial splitting of clusters for *K* = 6 as well in S5 Fig. On the other hand, for clustering results using *K* < 4 in S2 and S3 Figs, we did not see clear patterns. Therefore, we selected *K* = 4 as the number of clusters for this dataset, and all discussions hereafter are based on the results from this choice.

**Fig 4:**
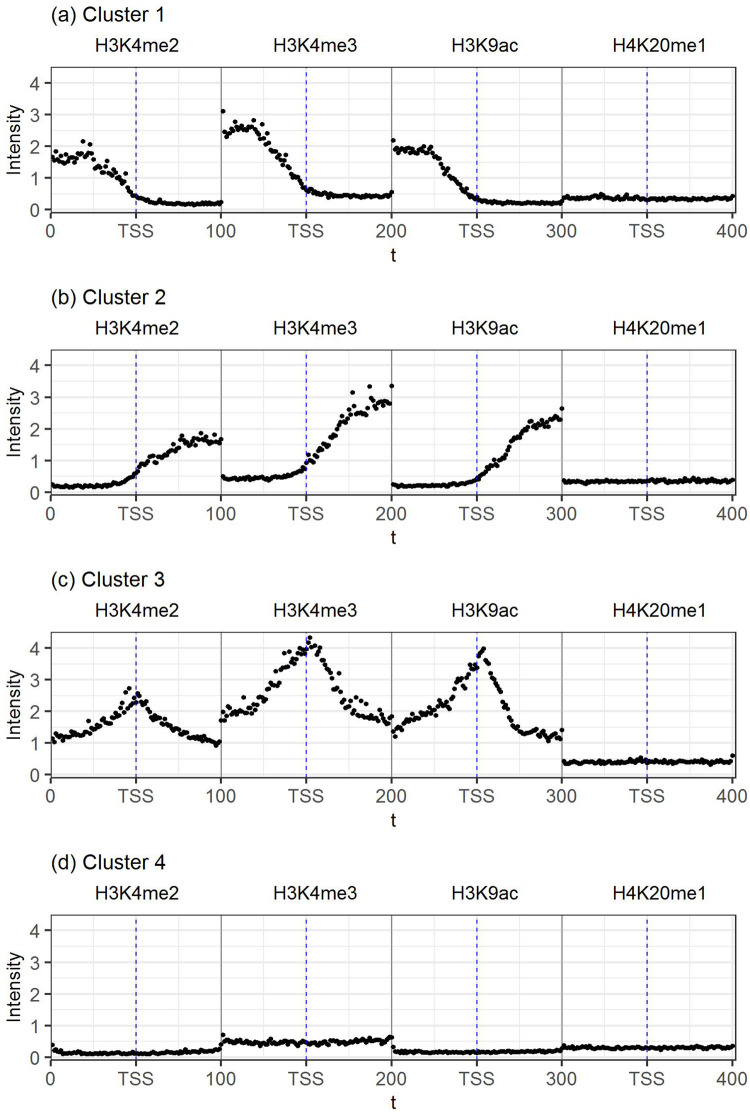
The four combinatorial histone modification patterns over four histone marks. (A) Cluster 1. (B) Cluster 2. (C) Cluster 3. (D) Cluster 4. For each cluster, the points represent the mean intensity counts for all profiles in the cluster.

To uncover the characteristic pattern for each cluster, we found the mean intensity counts and the corresponding smooth curve from the profiles belonging to that cluster. The results are shown in Fig 4, from which four distinct patterns are clearly seen. For cluster 1, we can see that the modification levels for the first three histone marks, H3K4me2, H3K4me3, and H3K9ac, are increased in the region upstream of the TSS, with the level reduces to the baseline level at the TSS. For cluster 2, the results are exactly the opposite: the increased modification levels for the first three histone marks are observed downstream of the TSS, from the baseline level at TSS to the highest level toward the end of the promoter region. For cluster 3, a symmetrical unimodal peak pattern with the peak marking the TSS is observed, again having the same pattern for each of the first three histone marks. Finally, for cluster 4, the pattern stays at the baseline level, indicating that this cluster captures inactive genes. Most interestingly, for H4K20me1, the modification levels stay at the baseline for all 4 clusters, supporting those results in the literature indicating that H4K20me1 does not mark gene promoters.

Comparing to the results in Heintzman et al. [1] and Taslim et al. [29] where *K* was also chosen to be 4, we can see that the last pattern signifying inactive gene promoters in our result matches those in the two papers. The cluster with unimodal peaks matches one of the clusters in Heintzman et al. [1] for H3K4me3, while the cluster with increased modification levels downstream of the TSS also matches, to some extent, another cluster for H3K4me3 [1]. On the other hand, none of the patterns in Taslim et al. [29] for H3K4me2, which hypothesized a bimodal pattern, match ours, possibly due to the more restrictive nature of their model. Similarly, the PVP patterns enforced by [5]are also not observed in our results.

### Putative promoters and their characteristics

**Fig 5:**
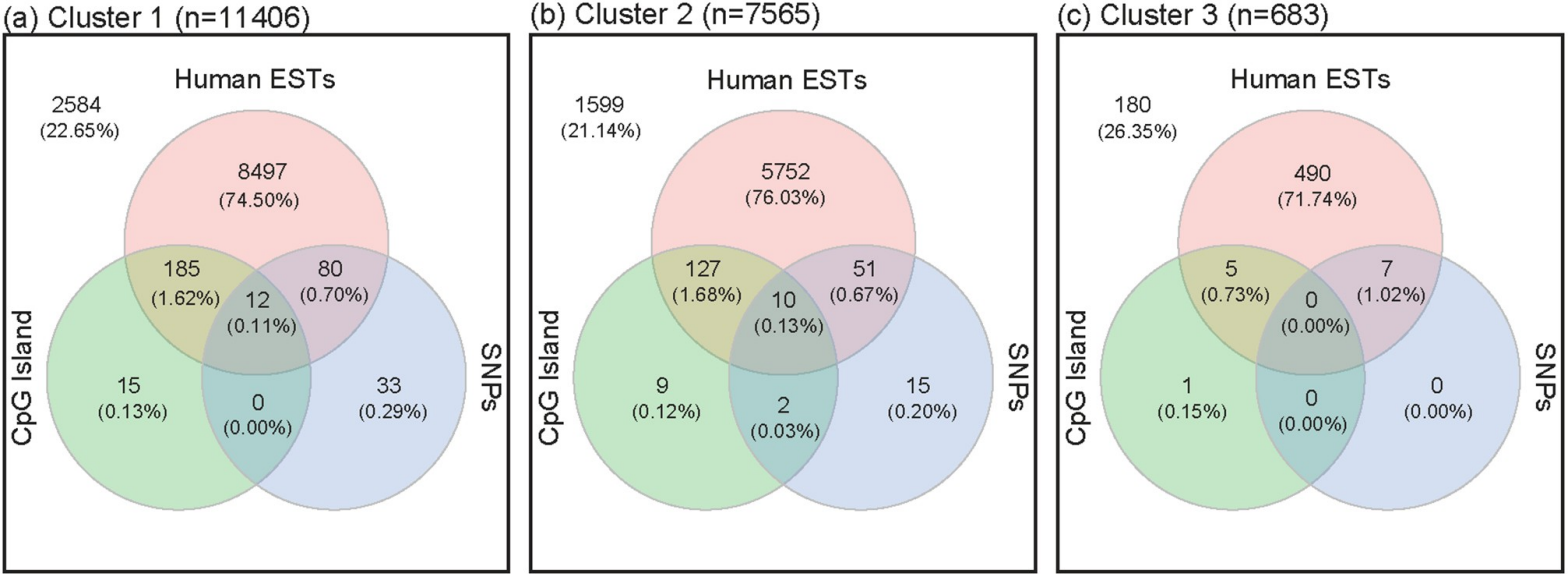
Venn diagrams of correlated region that overlap with Human ESTs, CpG Island or common SNPs. (A) Cluster 1 (n = 11406). (B) Cluster 2 (n = 7565). (C) Cluster 3 (n = 683). The number (%) outside of the circles indicate the putative promoter regions not overlapped with any of the three annotations.

Since the characteristic pattern of the last (4th) cluster signifies inactive genes, it was not used to scan for putative novel or alternative promoters throughout the genome. For characteristic patterns 1-3, we identified a total of 11406, 7565, and 683 putative promoters, respectively. To better understand the features of the regions identified, we seek to quantify their overlaps with ESTs (often used in gene discovery), CpG island (which may mark promoters), and common SNPs (which may serve as cis-regulator in a promoter region). To do so, we downloaded the elements for these three annotations from the UCSC Table Browser (https://genome.ucsc.edu/cgi-bin/hgTables). The Venn diagram in Fig 5 shows various intersections of these three annotations with the identified putative promoters. Overall, 77.8% of the putative promoters are overlapped with at least one annotation, seen evenly across the three clusters. Specifically, for the putative promoters with the cluster 1 binding pattern, 77.35% of them overlap with at least one of these annotations. For clusters 2 and 3, the percentages of overlaps with at least one of the three annotations are similarly high, at 78.86 and 73.56%, respectively. It is not surprising to see that most of the overlaps are accounted for by ESTs, although we also see a non-negligible number with CpG in clusters 1 and 2, in the order of hundreds. There are also quite a number of overlaps with common SNPs for clusters 1 and 2. However, for cluster 3, there are just a handful.

## Discussion

In this paper, we propose a novel two-step procedure to characterize the combinatorial patterns for four histone modifications, H3K4me2, H3K4me3, H3K9ac, and H4K20me1, in known gene promoters with high quality data. This is not a new problem, as a number of methods have been proposed to study the patterns in known promoters and predict new and alternative one. Nevertheless, this problem continues to be of interest as there are still unsettled issues in understanding the role of chromatin modifications in gene activation, especially the joint effect of modification of multiple histones. Although our analysis focuses on uncovering the underlying distinctive patterns of four specific histones as an example, the two-step procedure may be adapted and applied to other investigations where the goal is to characterize a heterogeneous population using data of high dimensionality.

Since existing analytical tools are viewed as lacking robustness [5], further investigation is warranted. Our current work is an attempt to provide a more robust approach to address some of these issues. Further, our proposed method is unbiased, and it also differs from existing methods in that it makes efficient use of the available data. More specifically, it is robust as it does not make any assumptions about the general patterns of modifications; rather, it lets the data “speak”. It makes efficient use of the data as it reduces noise by using a dimension reduction technique and it further accounts for spatial correlation, with the latter not accommodated in some other dimension reduction methods, such as non-negative matrix factorization. More importantly, by utilizing the uncorrelated property of the scores, we are able to formulate a mixture procedure to uncover several distinct binding patterns. Although our work is not the first to utilize the Karhunen-Loéve expansion for dimension reduction, in our proposed procedure, we take things one step further by using the uncorrelated property of the scores from the expansion to study binding pattern heterogeneity. We are able to utilize existing software for carrying out each of the steps in our two-step procedure to avoid “reinventing the wheel”. However, we would like to point out that, even though tools exist for each of the steps, using them in this combination for addressing the epigenetic problem in this paper is novel.

The results from analyzing the four histone modification levels from the human B-lymphoplastoid cell line show that our methodology has not only confirmed certain patterns already described in the literature, but also recovered unique patterns that have not been seen previously. Most interestingly, the three highly distinguishable patterns, increased modification levels before the TSS, after the TSS, and around the TSS, are shared among three histone marks, H3K4me2, H3K4me3, and H3K9ac. These results confirm the role of these three histones in marking active gene promoters. On the other hand, despite more recent results showing that actively transcribed genes are also characterized by high level of H4K20me1 in the promoters [15], our results for the B-lymphoblastoid cells do not confirm such findings; rather, our finding lends support to earlier results showing that H4K20me1 do not mark the promoter of active genes [38]. Indeed, among all four clusters of profiles, the H4K20me1 levels are never increased in the promoter regions.

Despite encouraging results, several issues, limitations, and alternative approaches deserve further discussions. First, it is an important, yet a difficult, task to determine the number of components in the mixture distribution in the second step of the two-step procedure. Although standard methods such as Akaike information criterion or Bayesian information criterion are frequently used, they may over or under estimate the number of patterns if there are substantial partial overlaps of individual profiles among the clusters. Therefore, in our analysis, we devised a procedure for determining the number of clusters by considering the distinctiveness of the clusters across the heatmaps. This procedure needs to be performed for each application, especially when different histone marks are considered or when the procedure is applied to a different problem with the same goal of uncovered multiple patterns in a heterogeneous population, as the optimum *K* may be different.

The threshold *α* for declaring significant correlation with a characteristic pattern to identify putative and alternative promoters also deserves further discussion. We have considered several *α* levels, including 0.3, 0.4, and 0.5. As we pointed out earlier, a higher *α* level will lead to a higher confidence in a potential novel promoter, but the number of identifications will be smaller. As can be seen from the heatmap (Fig 3) and the characteristic patterns (Fig 4), although the overall patterns are very clear and the profiles within each cluster appear to be homogeneous, there is still a fair amount of variability within each cluster. Therefore, when we computed the correlation of the observed profile for each promoter with the characteristic patterns, we obtained distributions with substantial variability. By setting *α* = 0.4, the majority of the known promoters in our set have a correlation exceeding this level with their respective characteristic pattern; yet the promoters falling below this threshold have less similarity with their characteristic curves and thus their clustering may not be as confident. On the other hand, the other *α* levels considered either led to too many known promoters below the threshold or too many promoters whose observe data do not exhibit a consistent pattern as the characteristic one. Thus, we decided to use *α* = 0.4 as the threshold for potential novel and alternative promoters.

To assess the biological significance of the predicted novel and alternative promoters, we considered three commonly used types of data, ESTs, CpG islands, and common SNPs, to provide corroborating evidence. Other data may also be used to provide further biological insights, such as the CTCF protein binding and DNAse hypersensitivity assay, although they were not considered in this study given our main objective of proposing a novel two-step procedure for analyzing high-dimensional data in a heterogeneous population. In the literaure, DNA methylation-associated features have also been used to predict promoters [44], which differs from this work as we focus on using histone methylation and acetylation data.

We also note that the idea of combining significance windows may lead to a large significance region. If a smaller region is desired (for one exceeding a certain length), then it may be reasonable to consider shorten the last, the first, or both significance windows, say by half. For instance, in the schematic profile provided in Fig 2A, the significance region may be shorten to 14701 bp—27300 bp instead of the original 9701 bp—32300 bp. Finally, two aspects of the procedure for determining the cluster membership also deserve further discussion. First, there may be situations where the cluster that has the highest peak may be due to a single (or only a couple of) significance window (representing a “spike” and potentially a false positive); thus, a criterion that not only uses the “height” but also the “width” would be worth considering. In our analysis, all our peaks were broad enough that we only considered the “height” in our criterion. Second, it is also possible to consider shortening the significance window at this stage. In the example in Fig 2D, the first significance region may be shorten from 9701 bp—40000 bp to 14701 bp—35000 b.p.

## Supporting information

S1 Fig An example of concatenated data. (PDF)

S2 Fig Heatmap of clusters for K = 2. (PDF)

S3 Fig Heatmap of clusters for K = 3. (PDF)

S4 Fig Heatmap of clusters for K = 5. (PDF)

S5 Fig Heatmap of clusters for K = 6. (PDF)
