# Supplementary material for: Characterization of histone modification patterns and prediction of novel promoters using functional principal component analysis
Source: PLoS One. 2020 May 27;15(5):e0233630. doi: 10.1371/journal.pone.0233630 (PMC7252632; doi:10.1371/journal.pone.0233630)
Supplement: S1 Fig — (PDF) [file pone.0233630.s001.pdf]

S1 Fig.

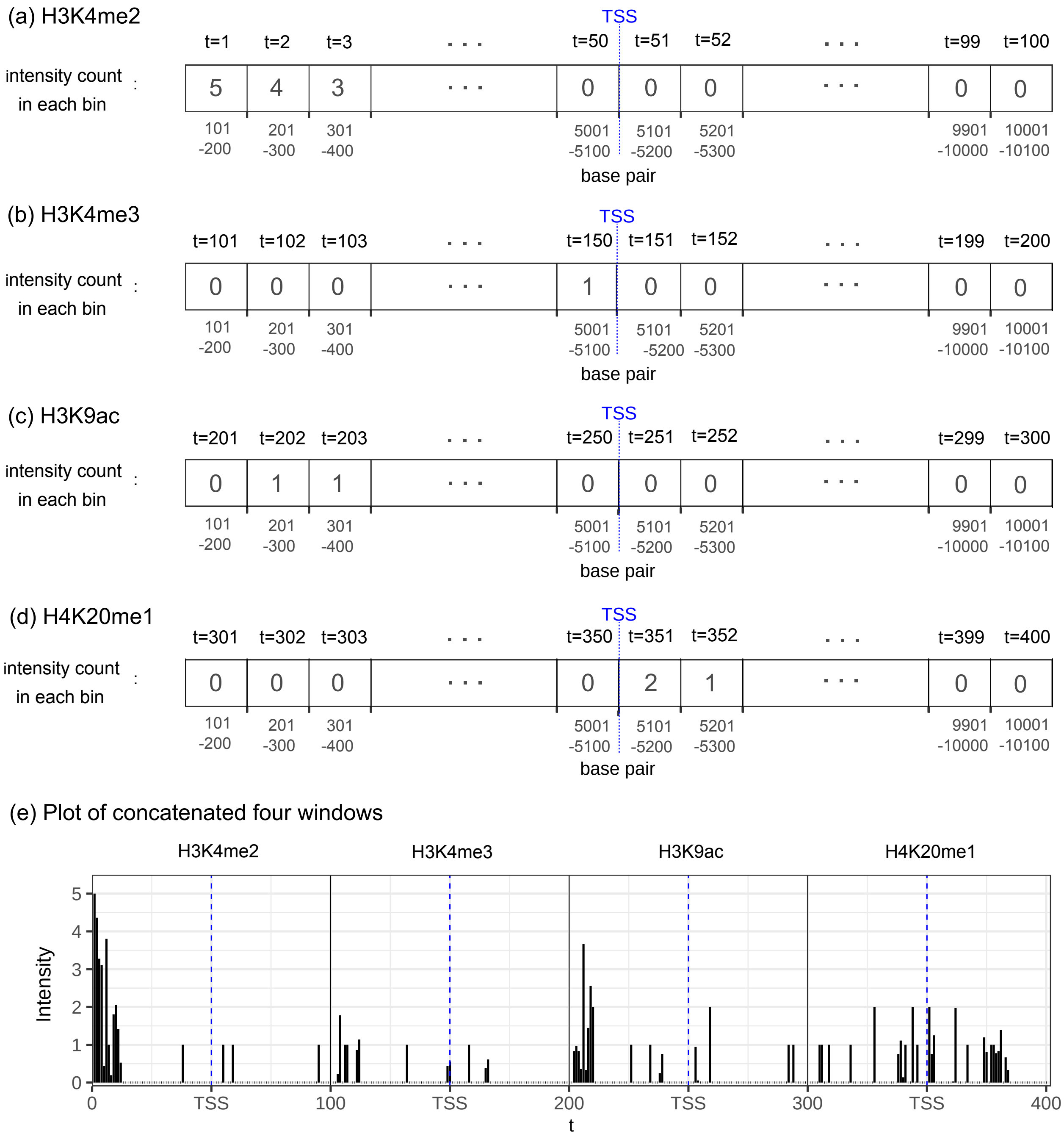

An example of concatenated data. A window with 100 bins is created from 10 kb regions around TSS in each Histone. (A) A window for H3K4me2. (B) A window for H3K4me3. (C) A window for H3K9ac. (D) A window for H4K20me1. (E) Plot of concatenated four windows.
