# Supplementary material for: Characterization of histone modification patterns and prediction of novel promoters using functional principal component analysis
Source: PLoS One. 2020 May 27;15(5):e0233630. doi: 10.1371/journal.pone.0233630 (PMC7252632; doi:10.1371/journal.pone.0233630)
Supplement: S2 Fig — (PDF) [file pone.0233630.s002.pdf]

S2 Fig.

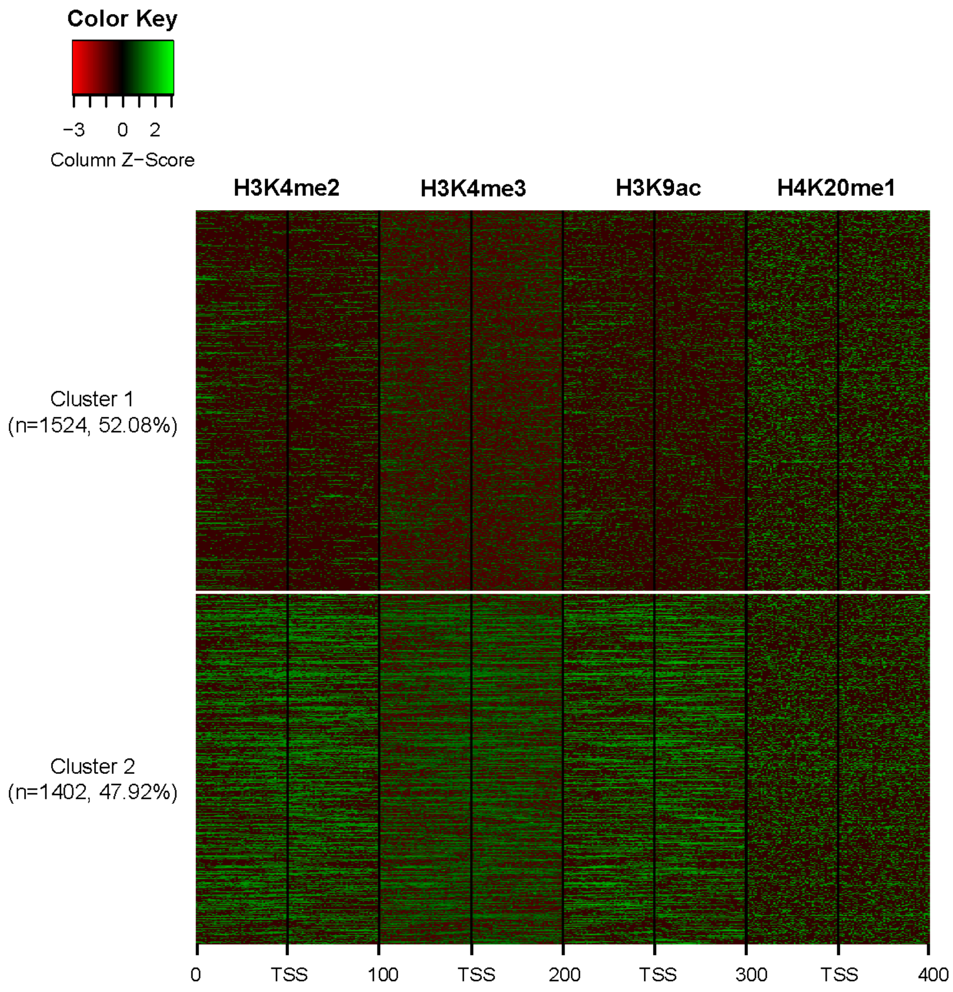

Heatmap of clusters for K = 2. Each row represents one profile over all four histone marks. Plotted are the color representation of the z-scores of the log-transformed intensity counts.
